# *Ad libitum* caffeine consumption, cognitive performance, and sleep in special forces soldiers during a 96-h combat exercise

**DOI:** 10.3389/fnins.2024.1419181

**Published:** 2024-06-21

**Authors:** David Erez, Harris R. Lieberman, Ido Baum, Itay Ketko, Daniel S. Moran

**Affiliations:** ^1^The Department of Health Systems Management, Ariel University, Ariel, Israel; ^2^Institute of Military Physiology, IDF Medical Corps, Tel Hashomer, Israel; ^3^Military Nutrition Division, U.S. Army Research Institute of Environmental Medicine (USARIEM), Boston, MA, United States

**Keywords:** sleep deprivation, caffeine, cognitive performance, mood, risky behaviors, military operations

## Abstract

**Introduction:**

This observational study investigated the effects of sleep deprivation and ad libitum caffeine consumption on cognitive performance, risk behavior, and mood among 28 Israeli Special Forces (SF) soldiers (mean age: 20.57 ± 0.92 years) during a 96-hour combat exercise.

**Methods:**

Actigraphy was used to monitor sleep and activity; cognitive function, risk-taking propensity, mood states, and self-reported sleepiness were assessed using the Psychomotor Vigilance Task (PVT), Evaluation of Risks Scale (EVAR), Profile of Mood States (POMS), Karolinska Sleepiness Scale (KSS); and caffeine consumption by questionnaire at 0, 50, and 96 hours. For analyses, participants were divided into Low (<400 mg) and High (≥400 mg) caffeine consumption groups.

**Results:**

The soldiers hiked 108.5 ± 0.52 km and slept for 12.7 ± 0.5 h, with a notable transition from multiple short sleep epochs in the initial 50 hours to a consolidated 5-hour sleep period subsequently. In the High caffeine group, PVT reaction time was faster (*p* = 0.024) compared to the Low caffeine group, with fewer premature response errors (*p* = 0.026). However, this group showed increased risk-taking (*p* = 0.037), particularly reduced Self-Control (*p* = 0.010). No significant impact of *ad libitum* caffeine intake on mood was observed. However, degradation over the course of the exercise in both groups in mood states, including anger, fatigue, tension, and vigor, was noted (*p* < 0.05). KSS scores increased significantly at 50 and 96 h (*p* < 0.001).

**Discussion:**

These results suggest that while caffeine enhances cognitive function, its ad libitum consumption did not consistently improve these measures in this cohort of SF soldiers. The study highlights the complex relationship between sleep deprivation and caffeine intake and their combined effects on soldiers’ cognitive and behavioral functions, indicating a need for evidence-based caffeine use guidelines for using caffeine in military settings.

## 1 Introduction

Caffeine, a xanthine alkaloid, is one of the most commonly consumed psychoactive substances worldwide (Weinberg and Bealer, 2004). Known primarily for its stimulating properties, caffeine exerts its effects by antagonizing adenosine receptors, which play a crucial role in sleep regulation and can also alter cerebral blood flow (Zwyghuizen-Doorenbos et al., 1990; Fredholm, 1995; Porkka-Heiskanen, 1999). During periods of sleep deprivation due to voluntary or involuntary sleep loss, caffeine’s ability to alleviate fatigue and enhance alertness has been a focal point of research, especially its influence on cognitive performance and mood modulation (McLellan et al., 2016; Patocka et al., 2019).

Sleep deprivation occurs when the quantity, quality, or timing of sleep is insufficient to maintain optimal brain function (Bonnet, 2011). It has been categorized into two types: total and partial sleep deprivation (Durmer and Dinges, 2005). Total sleep deprivation is continuous wakefulness exceeding the standard sleep–wake cycle. It has been sub-categorized into long-term total sleep deprivation (≥ 45 h) and short-term total sleep deprivation (<45 h). Partial sleep deprivation or sleep restriction is at least one night of interrupted or reduced sleep (<7 h/24 h). When this pattern persists for more than a week without adequate recovery sleep, it is termed chronic partial sleep deprivation (Durmer and Dinges, 2005).

The relationship between sleep deprivation and cognitive performance is dependent on the duration of sleep deprivation. Extended wakefulness beyond a threshold leads to lapses in alertness, suggesting a neurobiological deficit accumulating over time (Dinges et al., 1997; Åkerstedt et al., 2003; Balkin et al., 2004). Consequently, sleep deprivation impairs psychomotor vigilance, attention, reaction time, and memory (Belenky et al., 2003; Slutsky-Ganesh et al., 2017; Summers et al., 2021). This cognitive degradation can have significant consequences in high-stakes environments, such as the military, where rapid and accurate decision-making is crucial under stressful conditions (Sun et al., 2020; Paśko et al., 2022, for a review, see McLellan et al., 2004).

The profound effects of sleep deprivation on cognitive performance and mood have significant implications for active-duty military personnel, who often operate under conditions of partial sleep deprivation (Lieberman et al., 2005; Eliyahu et al., 2007; Szivak and Kraemer, 2015). These conditions can markedly impair cognitive, emotional, and physical capabilities, including deficits in marksmanship, physical performance, decision-making, and risk-taking behavior (McLellan et al., 2005; Kamimori et al., 2006; Grandou et al., 2019). Special Forces (SF) soldiers, in particular, face more rigorous and sustained operational demands compared to regular soldiers, pushing the boundaries of human endurance both physically and cognitively (Banderet et al., 1981; Castellani et al., 2006; Lieberman et al., 2006). Despite this, chronically sleep-deprived soldiers often mistakenly believe they can function optimally with minimal sleep by using caffeine (Bukhari et al., 2020).

Caffeine’s neurochemical effects influence cognitive processes and decision-making (Killgore et al., 2008b, 2011). Zhang et al. (2023) found that sleep deprivation increased impulsivity and emphasized the complex relationship between sleep and cognition, especially in high-stress settings like those of SF soldiers. While sleep deprivation heightens risk-taking behavior, caffeine intake may mitigate this effect, though it can also lead to overconfidence (Carvey et al., 2012; Cappelletti et al., 2015; Doepker et al., 2016), resulting in potentially risky decisions in combat situations (Vital-Lopez et al., 2018).

Although caffeine can exacerbate the effects of stress on risk-taking behaviors, it remains an effective countermeasure for sleep deprivation. Studies with military and civilian populations demonstrate that caffeine improves reaction time, reduces response lapses, and maintains motor planning and coordination (Brice and Smith, 2001; Crawford et al., 2017). However, it is important to note that caffeine does not restore normal functioning during sleep deprivation and cannot substitute for adequate restorative sleep (Killgore and Kamimori, 2020).

Furthermore, caffeine’s impact on mood states varies depending on the dosage consumed. Low doses of caffeine, typically less than 200 mg per day, are associated with improved mood and reduced symptoms of depression (Lieberman et al., 1987; Smit and Rogers, 2000; Smith, 2002; Haskell et al., 2005). Moderate doses, ranging from 200 to 400 mg per day, also yield positive mood effects but may induce negative side effects in sensitive individuals (Lieberman et al., 2002). Conversely, high doses of caffeine, exceeding 400 mg per day, can lead to a variety of negative mood states (Evans and Griffiths, 1999; Lieberman et al., 2002).

Finding the optimal dose and timing of caffeine consumption is crucial for individuals like SF soldiers, medical personnel, and long-haul truck drivers who use caffeine to enhance their performance. This optimization aims to achieve cognitive benefits without causing adverse mood effects that could potentially compromise interpersonal relationships, team cohesion, and overall mission success.

### 1.1 Study rationale, aim, and objective

Research has shown that controlled doses of caffeine can enhance cognitive function, including vigilance, learning, memory, and mood state, even in challenging circumstances (Lieberman et al., 2002; McLellan et al., 2016). However, the consequences of unregulated caffeine consumption during prolonged combat operations under conditions of sleep deprivation remain understudied. Therefore, this study investigated the interaction between accumulated sleep deprivation and *ad libitum* caffeine consumption on the cognitive performance, risky behaviors, mood states, and subjective sleepiness of Israeli SF soldiers during a 96-h combat exercise. The objective was to assess the effects of accumulated sleep deprivation and *ad libitum* caffeine consumption on the cognitive status measures and identify strategies to optimize SF soldier performance and well-being.

## 2 Methods

### 2.1 Participants

Twenty-eight male SF soldiers from a single Israel Defense Forces (IDF) unit volunteered to participate in this prospective observational study. The IDF Medical Corps and the Sheba Medical Center Institutional Ethics Review Board approved the study (ethics board approval number 5921–19). All subjects were briefed thoroughly about the research, and researchers addressed any questions or concerns before soldiers individually consented to participate in writing before any study-related activities. The soldiers were medically cleared by the unit physician, ensuring they had no chronic illness, medication use, or musculoskeletal injuries that could hinder exercise completion. No further inclusion or exclusion criteria were applied. The mean ± SEM age of the volunteers was 20.6 ± 0.2 years; their height and weight were 175.8 ± 1.1 cm and 70.7 ± 1.6 kg, respectively. Therefore, they provided a homogenous cohort. The volunteers had completed 16 months of their 36-month compulsory military service.

### 2.2 Study design and procedures

This prospective observational study monitored and analyzed the impact of sleep deprivation and *ad libitum* caffeine consumption on cognitive performance, propensity for risky behaviors, mood states, and sleep of Israeli SF soldiers during a 96-h combat exercise.

Recruitment and baseline evaluations were conducted a week before the exercise initiation. Anthropometric measurements and demographics were collected. At that time, subjects were trained and practiced the cognitive performance measures, the propensity to take risks questionnaire, and mood state assessments. Following recruitment and baseline evaluations, participants returned to their unit. They continued their usual military duties and sleep/wake schedules, which allowed for at least 7 h of regulated sleep per night. The 96-h exercise, including collecting dependent measures at 0, 50, and 96 h, commenced 1 week later.

### 2.3 SF combat exercise

Israeli SF soldiers are among the most highly trained soldiers in the IDF. The SF soldiers operate in small, flexible teams, specializing in guerrilla warfare, counterterrorism, special reconnaissance, and direct military action in complex and diverse terrains (IDF, 2022). Basic and advanced SF training lasts approximately 16 months and is designed to bring recruits to their optimal mental and physical state to prepare them for operational service. Their training culminates in an arduous, physically and cognitively demanding 96-h exercise that simulates various combat and special operations scenarios. Throughout the exercise, soldiers must complete their operational mission objectives while performing extensive long-range navigation with heavy loads in extreme geographical and weather conditions while coping with sleep deprivation. Soldiers require high levels of motivation and determination to complete this exercise and successfully graduate from SF training. The exercise took place in the hilly terrain of Northern Israel during September 2019, in weather conditions that ranged from a mean daytime temperature of 28.0°C (82.4°F) with 57.0% humidity to a mean nighttime temperature of 18.0°C (64.4°F) with 50.5% humidity.

### 2.4 Materials

Cognitive performance measures assessing reaction time and vigilance were conducted on IBM-compatible laptop computers (Lenovo ThinkPad T420; United States). Pen-and-paper questionnaires were administered to measure propensity for risky behaviors, mood states, sleepiness, and *ad libitum* caffeine consumption.

#### 2.4.1 Reaction time

The Psychomotor Vigilance Task (PVT) is a well-established tool for assessing reaction time, providing reliable measures of daytime fatigue and operational stress on cognitive performance (Dinges and Powell, 1985; Balkin et al., 2004). In this study, a 10-min version of the PVT was administered using a laptop at baseline and at predetermined intervals throughout the study. Participants were instructed to respond to visual stimuli presented on a blank laptop screen by pressing the space bar with their dominant index finger as quickly as possible. The stimuli appeared at random intervals, ensuring the task required sustained attention and vigilance. For data analysis, three primary metrics were recorded: mean reaction time (calculated as 1/reaction time * 1,000 to standardize the units), the number of response lapses (defined as reaction times exceeding 500 milliseconds), and the number of premature responses (responses occurring before the stimulus appeared). These measures have previously been used in sleep and cognitive performance research and are known for their sensitivity to the effects of sleep deprivation and cognitive workload (Dinges and Powell, 1985; Kamimori et al., 2006; Lim and Dinges, 2008; Basner and Dinges, 2011).

#### 2.4.2 Propensity for risky behaviors

A Hebrew pen-and-paper version of the Evaluation of Risks Scale (EVAR) was utilized to assess both state and trait aspects of risk propensity among participants (Killgore et al., 2006, 2008a). The assessment was administered to capture the subjects’ propensity for risk-taking behaviors. The EVAR scale comprises 24 items, each presented on a visual analog scale. Participants were asked to mark a point on the line that best represented the degree to which they felt each statement reflected their attitude. The EVAR scale is designed to measure five distinct factors of risk-taking propensity: Self-Control, Danger Seeking, Energy, Impulsiveness, and Invincibility. These factors provide a comprehensive assessment of the various dimensions of risk behavior. A total risk-propensity score was derived from the aggregate of all 24 items, offering a holistic view of the participants’ inclination toward risk-taking. This method has been shown to reliably distinguish between different levels of risk propensity in various populations (Sicard et al., 2001; Killgore et al., 2006, 2008a; Beckner et al., 2023).

#### 2.4.3 Mood states

Mood states were assessed using the Hebrew version of the Profile of Mood States (POMS) pen-and-paper questionnaire, a widely recognized tool for evaluating mood (McNair et al., 1971; Netz et al., 2005). Participants rated 28 mood-related adjectives on a scale from 0 (“not at all”) to 4 (“very much”), reflecting the intensity of their current mood. These adjectives were grouped into six sub-scales: five negative mood states (Tension, Depression, Anger, Fatigue, and Confusion) and one positive mood state (Vigor). A summary Total Mood Disturbance (TMD) score was calculated by summing the scores of the negative sub-scales and then subtracting the Vigor score. The resulting TMD scores range from −30 to 200, with lower scores indicating a more favorable mood state. The POMS has been used in various settings, including military populations, to assess the impact of stressors on mood (Penetar et al., 1993; Meney et al., 1998; Lieberman et al., 2006; Fuller et al., 2021; Beckner et al., 2023).

#### 2.4.4 Sleep and activity

Sleep and activity were measured using actigraphy (ActiGraph GT1M watch), with data analyzed via ActiLife™ Data Analysis Software (Version 4.0) [Software], ActiGraph, LLC. Actigraphy is a widely accepted method to assess and quantify sleep parameters and motor activity over extended periods of time in nonclinical settings (Ancoli-Israel et al., 2003). This method is particularly useful in field settings where traditional polysomnography (PSG), the gold standard for sleep measurement, is not feasible (Smith et al., 2018). Actigraphy has been validated against PSG for detecting wakefulness and sleep, making it a reliable tool for assessing sleep quality and duration (Marino et al., 2013). Subjects continuously wore the ActiGraph™ GT1M watch on their non-dominant wrist. The actigraph was placed in the medium sensitivity mode and recorded data in 30-s epochs (ActiGraph, 2009).

#### 2.4.5 Subjective sleepiness

The Karolinska Sleepiness Scale (KSS) was employed to assess the subjective sleepiness of participants. The KSS is a 10-point Likert-type scale, ranging from 1 (“extremely alert”) to 10 (“extremely sleepy; I cannot keep awake”), which measures sleepiness over the last 10 min (Akerstedt and Gillberg, 1990). This scale has been validated through its close correlation with electroencephalogram (EEG) and behavioral variables, underscoring its high validity and reliability in sleepiness assessment (Kaida et al., 2006; Akerstedt et al., 2014).

#### 2.4.6 Caffeine consumption

Caffeine consumption was assessed using a questionnaire based on the instrument developed by Lieberman et al. (2012) and adapted for the Israeli context. The questionnaire was administered at baseline (0 h) and at the 50- and 96-h time points, focusing on the quantity and frequency of caffeine-containing products consumed *ad libitum* in the preceding 24 h. The questionnaire included a comprehensive list of 43 commonly consumed caffeine-containing products, reflecting dietary patterns observed in similar studies (Lieberman et al., 2012; Knapik et al., 2016; Caldwell et al., 2018). Participants used a standard food frequency questionnaire format to select from the listed products and could also write in any additional caffeine-containing items not included on the list. This method has been validated for its accuracy in capturing dietary intake (Thompson and Byers, 1994). For analytical purposes, these products were categorized into five groups: coffee-based beverages, tea-based beverages, colas and other caffeine-based sodas/soft drinks, energy beverages, and caffeine-containing supplements and gums. The data from these categories were aggregated to determine the overall caffeine intake in the preceding 24 h.

### 2.5 Statistical analyses

Descriptive statistics are presented as mean ± standard error of the mean (SEM). Before analysis, all continuous variables were assessed for normal distribution using the Shapiro–Wilk test. The mean difference (Δ) between groups is reported where relevant, using an alpha level (α) of 0.05 to ascertain statistical significance. Participants were categorized based on their caffeine intake during the exercise into two groups: a ‘Low’ consumption group (<400 mg) and a ‘High’ consumption group (≥400 mg), consistent with the methodology employed in prior research (Fine et al., 1994; Flaten et al., 2003). A two-way analysis of variance (ANOVA) was conducted to examine the primary effects of time (at intervals of 0, 50, and 96 h) and caffeine consumption, as well as their interaction, on variables including cognitive performance, risk-taking tendencies, mood states, and subjective sleepiness. *Post hoc* comparisons were performed using Tukey’s Honestly Significant Difference (HSD) test for unequal sample sizes to identify significant temporal variations between low and high across the 0, 50, and 96-h assessment periods for each dependent variable. Partial eta-squared (η^2^) expressed effect sizes for F statistics. The Partial η^2^ measured how much the independent variable explained the variance in the dependent variable, indicating its effect size in a controlled analysis. All statistical procedures were executed using IBM SPSS Statistics for Windows, Version 29.0.1.0 [Software], IBM Corp.

## 3 Results

### 3.1 Sleep and activity

**Figure 1:**
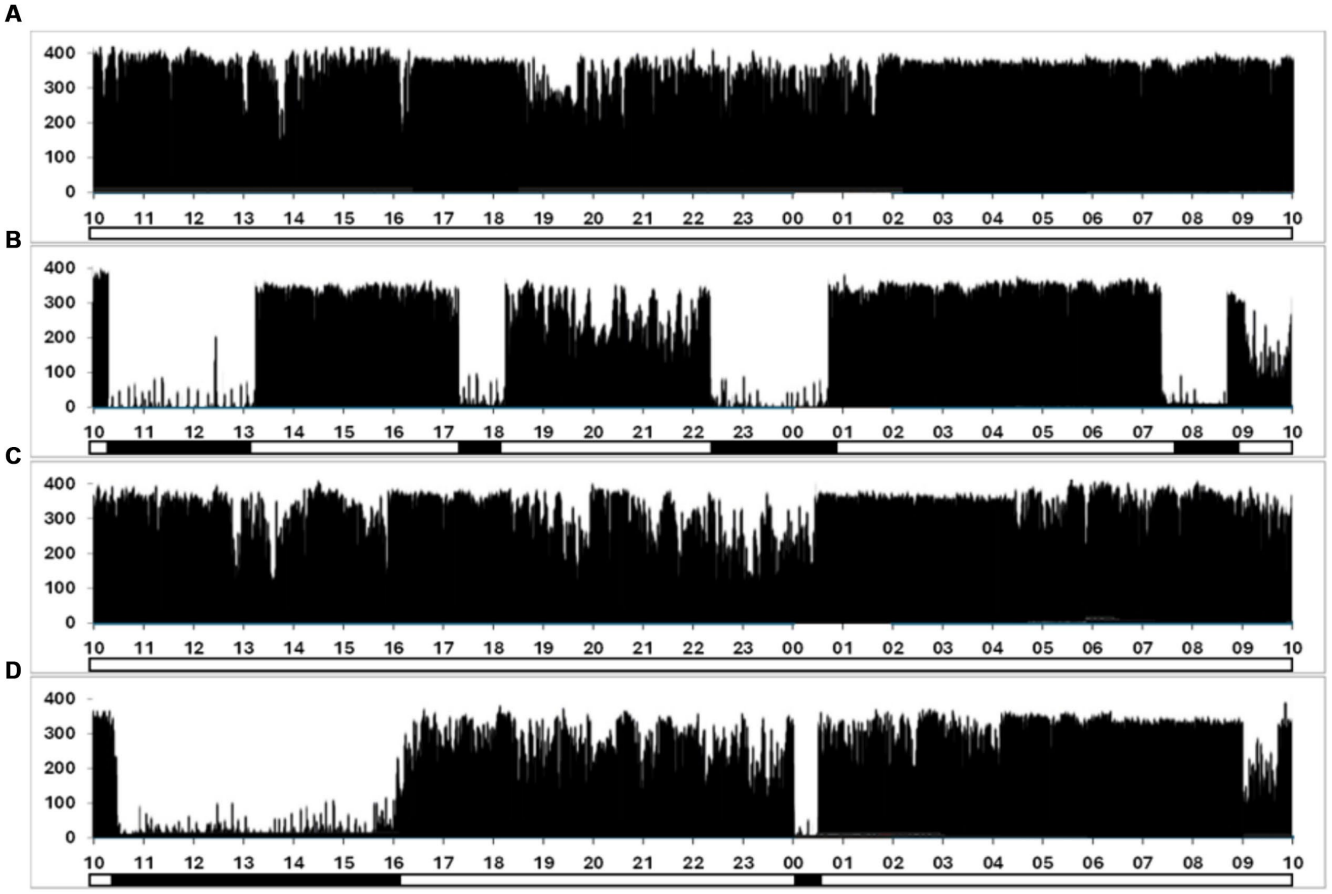
Representative actigraph data from one SF soldier was collected continuously over the 96-h exercise. **(A–D)** Correspond to a single 24-h period and plot the frequency of movements against time, measured in 60-s epochs. The y-axis quantifies movement counts, with the magnitude of the vertical line reflecting the detected movement count within each epoch. A secondary binary-coded axis beneath the x-axis indicates estimated sleep (solid bars) and wakefulness (clear segments) phases.

Actigraphic data revealed that subjects accumulated 12.7 ± 0.5 h (mean ± SEM) of sleep over the 96-h exercise, indicating significant sleep deprivation. During the first 50 h of the exercise, the subjects slept an average of 7.5 h, distributed over multiple epochs. From the 50-h mark until the completion of the exercise, subjects slept an average of 5.2 h. However, unlike the first 50 h, sleep during this second period was consolidated into a single sleeping period, as shown in Figure 1, representative actigraphy data from one SF soldier. During the 96-h field exercise, participants hiked a mean ± SEM distance of 108.5 ± 0.52 km, carrying loads up to 40% of their body weight.

### 3.2 *Ad libitum* caffeine consumption

During the 96-h exercise, all participants consumed caffeine *ad libitum*. Caffeine in various forms was obtained from standard rations, and soldiers carried additional food, beverages, and supplements. At the 0-h assessment point (baseline), there was a significant difference (*p* < 0.0001) between the Low (*n* = 21) and High (*n* = 7) caffeine groups, (mean ± SEM) 76.66 ± 17.41 versus 556.00 ± 66.68, respectively. While the overall mean caffeine consumption during the exercise was 146.64 ± 22.44, there was a significant difference between the Low and High caffeine groups, with the mean intake being 64.32 ± 10.18 for the Low and 525.56 ± 43.79 for the High consumption group (*p* < 0.0001) (Table 1). Of note, one subject recorded ingesting a single dose of 1,000 mg of caffeine in over-the-counter supplement pills, which was equivalent to an intake of 14.6 mg/kg of body weight.

**Table 1:** Caffeine consumption (mg) over the previous 24 h.

Hours	Low (mg) (*N* = 21)	High (mg) (*N* = 7)
0	76.66 ± 17.41	556.00 ± 66.68
50	55.42 ± 16.72	455.00 ± 50.12
96	60.39 ± 18.62	548.48 ± 66.85
24-h average	64.32 ± 10.18	525.56 ± 43.79

Caffeine consumption (mg) over the previous 24 h at three time points (0, 50, and 96 h) during a combat exercise. Data presented as mean ± SEM. Caffeine consumption “Low” (<400 mg), and “High” (≥400 mg).

### 3.3 Cognitive performance

#### 3.3.1 Mean RT

The analysis revealed a significant effect of caffeine consumption [*F*(1, 78) = 5.333; *p* = 0.024; η^2^ = 0.064], indicating faster mean RT for high caffeine consumers. There were no significant time effects [*F*(2, 78) = 0.416, *p* = 0.661; η^2^ = 0.011]. The interaction between time and caffeine consumption was not significant [*F*(2, 78) = 0.890; *p* = 0.415; η^2^ = 0.022] (Table 2).

**Table 2:** Cognitive performance, propensity to take risks, and mood states to high and low caffeine consumption during the 96-h exercise.

Variable	0 h (Low/High)	50 h (Low/High)	96 h (Low/High)	*F*(df)	*p*-value	Partial η^2^
**Psychomotor vigilance task**						
Mean reaction time (ms)	0.33 ± 0.01/0.32 ± 0.02	0.34 ± 0.01/0.31 ± 0.00	0.34 ± 0.01/0.32 ± 0.01	5.333 (1, 78)	0.024	0.064
Premature responses (%)	6.31 ± 1.16/5.14 ± 2.49	9.60 ± 1.58/4.02 ± 1.40	10.16 ± 1.47/3.89 ± 2.21	5.137 (1, 78)	0.026	0.062
Timeout responses (%)	4.84 ± 1.15/8.55 ± 4.84	8.51 ± 1.36/3.49 ± 1.26	9.20 ± 1.47/3.90 ± 2.21	1.396 (1, 78)	0.241	0.018
**Propensity to take risks**						
EVAR	12.86 ± 0.05/14.76 ± 0.31	11.93 ± 0.10/12.92 ± 0.64	12.02 ± 0.07/13.31 ± 0.56	4.494 (1, 78)	0.037	0.054
Self-control	71.50 ± 3.78/63.00 ± 14.38	82.12 ± 4.96/53.50 ± 18.24	74.95 ± 3.50/61.86 ± 9.21	6.945 (1, 78)	0.01	0.082
Danger seeking	52.92 ± 2.53/34.00 ± 4.14	62.76 ± 3.30/59.00 ± 8.55	60.14 ± 3.71/59.00 ± 7.69	5.649 (2, 78)	0.005	0.127
Energy	46.83 ± 2.45/35.75 ± 4.87	60.92 ± 2.84/52.25 ± 10.93	52.62 ± 2.84/52.57 ± 5.92	4.790 (2, 78)	0.011	0.109
Impulsiveness	86.58 ± 3.57/83.50 ± 6.44	88.08 ± 4.18/86.00 ± 6.88	92.29 ± 4.02/87.71 ± 8.56	0.302 (2, 78)	0.74	0.008
Invincibility	49.33 ± 2.79/31.50 ± 6.59	50.88 ± 3.66/56.00 ± 13.21	50.48 ± 4.14/52.86 ± 9.04	2.236 (2, 78)	0.114	0.054
**Profile of mood states**						
Total mood disorder	95.67 ± 2.33/95.75 ± 4.94	117.29 ± 2.15/119.00 ± 6.70	106.19 ± 1.65/108.57 ± 3.32	16.867 (2, 78)	<0.001	0.302
Anger	1.54 ± 0.60/0.25 ± 0.25	5.21 ± 0.74/7.75 ± 3.07	1.29 ± 0.44/3.00 ± 1.45	12.050 (2, 78)	<0.001	0.236
Depression	2.08 ± 0.65/1.75 ± 1.03	5.33 ± 0.96/4.00 ± 2.45	1.52 ± 0.47/2.43 ± 1.57	2.751 (2, 78)	0.07	0.066
Fatigue	2.42 ± 0.50/5.00 ± 1.73	11.46 ± 0.56/11.75 ± 1.84	10.33 ± 0.73/10.86 ± 1.39	29.709 (2, 78)	<0.001	0.432
Tension	3.25 ± 0.48/2.25 ± 0.75	1.96 ± 0.36/2.00 ± 0.71	0.62 ± 0.21/0.86 ± 0.46	5.727 (2, 78)	0.005	0.128
Vigor	13.62 ± 0.80/13.50 ± 2.53	6.67 ± 0.68/6.50 ± 2.06	7.57 ± 0.89/8.57 ± 1.32	13.081 (2, 78)	<0.001	0.251
**Subjective sleepiness**						
KSS	4.00 ± 0.36/3.75 ± 0.48	8.58 ± 0.24/7.50 ± 1.55	7.52 ± 0.37/6.57 ± 0.97	22.527 (2, 78)	<0.001	0.366

The effect of high and low caffeine consumption on cognitive performance, propensity to take risks, Profile of Mood States, and subjective sleepiness during a combat exercise at 0, 50, and 96 h. Data presented as mean ± SEM.

#### 3.3.2 Percent premature

The caffeine consumption effect was significant [*F*(1, 78) = 5.137; *p* = 0.026; η^2^ = 0.062], with high caffeine consumers making fewer premature responses. There was no significant Time effect [*F*(2, 78) = 0.174; *p* = 0.841; η^2^ = 0.004]. The Time*Caffeine Consumption interaction was not significant [*F*(2, 78) = 0.683; *p* = 0.508; η^2^ = 0.017; Table 2].

#### 3.3.3 Percent timeout

No significant effects were found for caffeine consumption [*F*(1, 78) = 1.396; *p* = 0.241; η^2^ = 0.018], or time [*F*(2, 78) = 0.047; *p* = 0.954; η^2^ = 0.001]. The interaction effect approached significance [*F*(2, 78) = 2.406; *p* = 0.097; η^2^ = 0.058; Table 2].

### 3.4 The propensity for risky behavior

#### 3.4.1 Overall propensity for risky behavior (EVAR)

The analysis revealed significant effects of caffeine consumption on EVAR [*F*(1, 78) = 4.494; *p* = 0.037; η^2^ = 0.054]. Participants with high caffeine consumption had a higher mean EVAR (13.465 ± 0.512) than those with low consumption (12.275 ± 0.231). However, no significant time effect [*F*(2, 78) = 2.701; *p* = 0.073] or time-caffeine interaction [*F*(2, 78) = 0.424; *p* = 0.656] was observed (Table 2).

#### 3.4.2 Self-control

Significant effects were found for caffeine consumption [*F*(1, 78) = 6.945; *p* = 0.010; η^2^ = 0.082] but not for time [*F*(2, 78) = 0.012; *p* = 0.988] or time-caffeine interaction [*F*(2, 78) = 0.834; *p* = 0.438]. Participants with low caffeine consumption reported higher Self-Control (76.192 ± 2.613) than those with high consumption (59.452 ± 5.790) (Table 2).

#### 3.4.3 Danger-seeking

There was a time effect [*F*(2, 78) = 5.649; *p* = 0.005; η^2^ = 0.127] for the mean Danger-Seeking score between the highest at 50 h (60.880 ± 4.200) and the lowest at 0 h (43.458 ± 4.200). The caffeine consumption [*F*(1, 78) = 3.031; *p* = 0.086] and time-caffeine interaction [*F*(2, 78) = 1.453; *p* = 0.240] were not significant (Table 2).

#### 3.4.4 Energy

Significant time effects were observed in Energy [*F*(2, 78) = 4.790; *p* = 0.011; η^2^ = 0.109]. Energy levels were higher at 50 h (56.583 ± 3.663) compared to 0 h (41.292 ± 3.663). Neither caffeine consumption [*F*(1, 78) = 2.753; *p* = 0.101] nor the interaction effect [*F*(2, 78) = 0.808; *p* = 0.450] showed significant differences (Table 2).

#### 3.4.5 Impulsiveness

The EVAR impulsiveness variable did not demonstrate significant differences for caffeine consumption [*F*(1, 78) = 0.346; *p* = 0.558], time [*F*(2, 78) = 0.302; *p* = 0.740], or their interaction [*F*(2, 78) = 0.019; *p* = 0.981]. The mean Impulsiveness scores remained consistent across time points and caffeine consumption levels (Table 2).

#### 3.4.6 Invincibility

The Invincibility variable showed no significant effects for caffeine consumption [*F*(1, 78) = 0.431; *p* = 0.513], time [*F*(2, 78) = 2.236; *p* = 0.114], or the interaction between the two [*F*(2, 78) = 1.768; *p* = 0.177; Table 2].

### 3.5 Mood states

#### 3.5.1 Total mood disturbance

A significant main effect of time was observed [*F*(2, 78) = 16.867; *p* < 0.001; η^2^ = 0.302], with TMD scores increasing significantly from 0 h to 50 h (Δ = −22.437; *p* < 0.001) and from 0 h to 96 h (Δ = −11.673; *p* = 0.001). Caffeine consumption did not significantly affect TMD scores [*F*(1, 78) = 0.220; *p* = 0.641], and the interaction effect between time and caffeine consumption was also non-significant [*F*(2, 78) = 0.054; *p* = 0.947; Table 2].

#### 3.5.2 Anger

Time significantly affected Anger levels [*F*(2, 78) = 12.050; *p* < 0.001; η^2^ = 0.236], with increases noted from 0 h to 50 h (Δ = −5.583; *p* < 0.001) and from 50 h to 96 h (Δ = 4.336; *p* < 0.001). No significant effect of caffeine consumption [*F*(1, 78) = 1.130; *p* = 0.291] or time-caffeine interaction [*F*(2, 78) = 1.429; *p* = 0.246] was found (Table 2).

#### 3.5.3 Depression

The effect of time on Depression scores was marginally significant [*F*(2, 78) = 2.751; *p* = 0.070; η^2^ = 0.066], with an increase from 0 h to 50 h (Δ = −2.750; *p* = 0.051) and a decrease from 50 h to 96 h (Δ = 2.690; *p* = 0.036). Caffeine consumption [*F*(1, 78) = 0.057; *p* = 0.812] and its interaction with time [*F*(2, 78) = 0.406; *p* = 0.668] did not significantly influence Depression scores (Table 2).

#### 3.5.4 Fatigue

There was a significant influence of time on Fatigue scores [*F*(2, 78) = 29.709; *p* < 0.001; η^2^ = 0.432], marked by increases from 0 h to both 50 h (Δ = −7.896; *p* < 0.001) and 96 h (Δ = −6.887, *p* < 0.001). Caffeine consumption had no significant impact [*F*(1, 78) = 1.673; *p* = 0.200], and the interaction effect of time and caffeine consumption was also not significant [*F*(2, 78) = 0.644; *p* = 0.528; Table 2].

#### 3.5.5 Tension

There was a significant time effect on Tension scores [*F*(2, 78) = 5.727; *p* = 0.005; η^2^ = 0.128], with an increase from 0 h to 96 h (Δ = 2.012; *p* = 0.002) and a decrease from 50 h to 96 h (Δ = −1.241; *p* = 0.046). No significant effect was observed for caffeine consumption [*F*(1, 78) = 0.216; *p* = 0.643] or the interaction between time and caffeine consumption [*F*(2, 78) = 0.546; *p* = 0.581; Table 2].

#### 3.5.6 Vigor

Vigor scores were significantly affected by time [*F*(2, 78) = 13.081; p < 0.001; η^2^ = 0.251]. There was a noted decrease in Vigor from 0 h to 50 h (Δ = −6.979; *p* < 0.001) and from 0 to 96 h (Δ = −5.491; *p* < 0.001). Neither caffeine consumption [*F*(1, 78) = 0.044; *p* = 0.834] nor its interaction with time [*F*(2, 78) = 0.134; *p* = 0.875] showed a significant effect (Table 2).

### 3.6 Subjective sleepiness

#### 3.6.1 KSS

The analysis revealed a significant effect of time on KSS scores [*F*(2, 78) = 22.527; *p* < 0.001; η^2^ = 0.366]. There was no significant effect of caffeine consumption [*F*(1, 78) = 2.284; *p* = 0.135] and no significant interaction between time and caffeine consumption [*F*(2, 78) = 0.242; *p* = 0.785; Table 2].

*Post hoc* analyses using the Tukey HSD test revealed significant differences in KSS scores between all-time points (0 h vs. 50 h, 0 h vs. 96 h, and 50 h vs. 96 h), with the highest sleepiness reported at 50 h. The KSS scores were significantly higher at 50 h (8.042 ± 0.464) compared to 0 h (3.875 ± 0.464) and 96 h (7.048 ± 0.375). The mean difference between 0 h and 50 h was −4.167 (*p* < 0.001), and between 0 and 96 h was −3.173 (*p* < 0.001), indicating increased sleepiness over time.

## 4 Discussion

Actigraphy revealed that subjects slept a cumulative mean time of approximately 12.7 h over the course of the 96-h exercise. This pattern of restricted sleep distributed over 96 h indicates short-term total sleep deprivation. This was particularly evident in the first 50 h, where subjects averaged 7.5 h of sleep, with sleep distributed over multiple short epochs. Notably, sleep patterns shifted from fragmented sleep in the initial 50 h to a consolidated 5-h sleep period thereafter. Additionally, the physical demands of the exercise were evident, as participants hiked an average of 108.5 km, carrying loads up to 40% of their body weight.

Participants were categorized into two distinct groups based on their caffeine consumption behavior (Low <400 mg versus High ≥400 mg). They were consistent throughout the training exercise, with differences indicating habitual caffeine use. The significant difference in caffeine consumption between the Low and High caffeine groups, with the former consuming an average of 64.32 mg compared to 525.56 mg in the latter, indicates differing reliance on caffeine use under conditions of sleep deprivation. This variation is noteworthy, given its significant impact on cognitive functions, suggesting its utility as a stimulant in challenging environments (Lieberman et al., 2002; Kamimori et al., 2005; McLellan et al., 2005, 2016). It is worth mentioning that individuals who consume high amounts of caffeine tend to develop some level of tolerance to its effects (Evans and Griffiths, 1992; Lara et al., 2019).

### 4.1 Impact of caffeine on cognitive performance during sleep deprivation

Our findings indicate that time did not significantly affect PVT performance or interact significantly with caffeine consumption. Several factors could explain these observations. One plausible explanation is that caffeine intake, even in the low caffeine group, may have mitigated the expected decline in PVT performance typically associated with sleep deprivation. Caffeine enhances alertness and cognitive function by antagonizing adenosine receptors, temporarily counteracting some cognitive impairments induced by sleep loss (Fredholm et al., 1999; McLellan et al., 2016). In our study, soldiers in both caffeine groups may have ingested sufficient amounts of caffeine to counteract sleep deprivation effects on PVT performance. This is supported by the significant positive impact of caffeine on Mean RT and Percent Premature Responses, especially in the high caffeine group.

A combined effect of caffeine intake and inherent resilience to sleep deprivation may explain the lack of significant temporal effects on PVT performance. Soldiers’ rigorous training may enhance their ability to cope with sleep loss and maintain cognitive function under adverse conditions (Lieberman et al., 2005). The high caffeine consumption group demonstrated improved Mean RT and Percent Premature Responses, highlighting caffeine’s role in sustaining performance. The lack of significant time effects suggests that participants’ baseline resilience, coupled with caffeine’s benefits, collectively buffered against the expected cognitive decline.

### 4.2 Caffeine consumption and risky behaviors

Our findings corroborate the literature on the effects of caffeine on risk-taking behaviors. While our study found that high *ad libitum* caffeine consumption increased general EVAR scores, Killgore et al. (2011) reported that caffeine administration reduced impulsivity and risk-taking behaviors. The discrepancy may be due to differences in study design, such as the regulated caffeine administration in Killgore et al. (2011) versus our study’s *ad libitum* consumption. Furthermore, high caffeine intake in our study may have led to overconfidence, potentially increasing risk-taking behavior (Carvey et al., 2012; Cappelletti et al., 2015; Doepker et al., 2016).

### 4.3 Mood states

Changes during the exercise were present for the Profile of Mood States (POMS). There was a marked degradation in Total Mood Disturbance, Anger, Depression, Fatigue, Tension, and Vigor over time. These variations occurred due to the intense nature of the exercise and were unrelated to caffeine intake, as caffeine consumption did not significantly impact these mood states. This has been observed in multiple studies of multi-stressor training exercises that include substantial sleep deprivation and have a greater impact than caffeine intake (Lieberman et al., 2005; Eliyahu et al., 2007; Szivak and Kraemer, 2015).

Lastly, Karolinska Sleepiness Scale (KSS) results indicated a significant increase in self-reported sleepiness over time, especially at the 50-h mark. This trend demonstrates the impact of prolonged wakefulness on soldiers’ perceived sleepiness, as the POMS Fatigue and Vigor scores demonstrated. While caffeine may enhance certain cognitive functions, it did not substantially counteract the adverse changes induced by extended periods of wakefulness.

### 4.4 Limitations

This research has several limitations. First, prospective observational studies lack the ability to establish causality, although they allow investigation into diverse outcomes where experimental manipulations may be unfeasible. The relatively small sample size and specific cohort (Israeli SF soldiers) limit the generalizability of the findings. However, using objective measurements and self-reported data helps mitigate self-report bias and increases the study’s relevance. The potential impact of some confounding variables was likely attenuated due to the highly homogeneous cohort created through SF soldiers’ rigorous selection and training regimen.

Second, *ad libitum* caffeine consumption could introduce individual bias. Future studies should control for caffeine dosage to understand its potential benefits and limitations better. Additionally, the scope of the current study did not include an examination of habitual caffeine consumption or the potential of subjects to develop tolerance or dependence on caffeine. Expectance of caffeine effectiveness could also be an important variable to account for in future research. While actigraphy is a widely accepted method for sleep assessment in non-clinical environments, it has limitations, such as underestimating wake-after-sleep onset (WASO) and overestimating total sleep duration (Sadeh, 2011). Moreover, in military contexts, periods of inactivity do not necessarily equate to sleep. Soldiers might remain motionless during tasks like surveillance or maintaining an ambush position, where they are cognitively engaged but physically stationary, potentially leading to overestimating sleep duration and quality in the data.

### 4.5 Implications for military operations and training

This study underscores the need for structured protocols assessing caffeine consumption within the context of military operations and training and other stressful environments. While controlled caffeine intake can alleviate cognitive impairments caused by fatigue, indiscriminate or unregulated use does not consistently offer these benefits. Moreover, a comprehensive review of the effects of caffeine consumption in healthy adults concluded that caffeine consumption up to 400 mg/day is not associated with adverse physiological or health risks (Nawrot et al., 2003).

Consequently, it is essential for the IDF and others to establish evidence-based caffeine consumption guidelines for its personnel. These guidelines should detail optimal dosing schedules, set maximum intake limits, and outline contraindications, thereby maximizing caffeine’s cognitive benefits while minimizing its adverse effects. A U.S. Department of Defense (DoD) ration component for caffeine delivery (the Military Energy Gum) contains 100 mg caffeine per piece with packaging guidance stating, “Chew one piece at a time for 5 min, and if you aren’t alert within 15 min, chew a second piece. Do not exceed 2 pieces in a 3-h time period or more than 8 pieces in 24 h” (a total daily dose of 800 mg) (The Consortium for Health and Military Performance, 2019). The U.S. DoD recommended position statement states, “…caffeine intake should be maintained to the daily 2–6 mg/kg per dose range or below 400 mg/day unless it is being used to sustain performance during extended wakefulness,” and official U.S. Navy policy states “…caffeine intake of 450 mg per day (3 to 4 cups of drip coffee) is the recommended maximum intake. When managed appropriately, caffeine use can aid in maximizing performance during…periods of sustained operations; however, the caffeine effect is maximized in individuals who are not habituated to its effect as regular users…” (The Consortium for Health and Military Performance, 2020). In the absence of controlled use, the risk of caffeine misuse increases, potentially diminishing its positive cognitive effects and increasing the likelihood of detrimental health outcomes (Nawrot et al., 2003). Therefore, formulating and implementing these guidelines should be a strategic priority, requiring prompt action from military medical authorities, command structures, and policymakers.

### 4.6 Broader implications beyond military settings

These findings also have significant implications for professions like emergency services and healthcare, where workers often face similar challenges of sleep deprivation and high-stress environments. Understanding how caffeine consumption might influence cognitive performance and decision-making in these settings could provide guidelines for stimulant use among these professions.

Furthermore, these findings are also relevant for occupations involving shift work or extended hours, such as in transportation or manufacturing industries. A comprehensive understanding of caffeine’s effects on sleepiness and cognitive functions could guide strategies for managing work schedules and rest periods to optimize alertness and minimize risks.

## 5 Conclusion

This study offers novel insights into the complex interplay between caffeine consumption and sleep deprivation in military combat settings. It demonstrates that caffeine consumption can mitigate some effects of sleep loss on cognitive abilities. However, its effectiveness is inconsistent and certainly not a substitute for adequate sleep. Adequate sleep remains crucial for maintaining peak cognitive function, physical health, and psychological well-being. Consequently, military policies should prioritize sleep as a cornerstone of operational readiness and performance, ensuring conditions conducive to sufficient and restorative rest for soldiers. This research paves the way for further studies to investigate the potential of regulated caffeine use—optimizing dosage and timing—as a potential measure to counteract the cognitive and behavioral consequences of sleep deprivation. Additional studies in diverse global military and civilian scenarios will be essential for developing comprehensive strategies that balance the need for alertness with the imperative of restorative sleep to enhance overall mission effectiveness and soldier welfare.

## Data availability statement

The data in this article cannot be shared publicly as they belong to the Israel Defense Forces and the Israel Ministry of Defense. Data sharing will be considered if the corresponding author receives a reasonable request and establishes an institution-to-institution data-sharing agreement.

## Ethics statement

The studies involving humans were approved by the IDF Medical Corps and the Sheba Medical Center Institutional Ethics Review Board. The studies were conducted in accordance with the local legislation and institutional requirements. The participants provided their written informed consent to participate in this study.

## Author contributions

DE: Data curation, Formal analysis, Investigation, Methodology, Software, Visualization, Writing – original draft. HL: Writing – review & editing. IB: Writing – review & editing, Data curation, Investigation, Resources, Software. IK: Writing – review & editing. DM: Investigation, Conceptualization, Funding acquisition, Project administration, Supervision, Validation, Writing – review & editing.

## Author disclaimer

The opinions or assertions contained herein are the private views of the authors. They are not to be construed as official or as reflecting the views of the Israeli Ministry of Defense and the Israel Defense Force, or the United States Army or Department of Defense. Any citations of commercial organizations and trade names in this report do not constitute an official Israeli Ministry of Defense and the Israel Defense Force or US Department of the Army endorsement or approval of the products or services of these organizations.
