# Supplementary material for: Ad libitum caffeine consumption, cognitive performance, and sleep in special forces soldiers during a 96-h combat exercise
Source: Front Neurosci. 2024 Jun 21;18:1419181. doi: 10.3389/fnins.2024.1419181 (PMC11224469; doi:10.3389/fnins.2024.1419181)
Supplement: Supplementary file 1 [file Data_Sheet_1.PDF]

Supplementary Material

Ad Libitum Caffeine Consumption, Cognitive Performance, and Sleep in Special Forces Soldiers during a 96-hour Combat Exercise

Figure 1. Representative Actigraph Data from one SF Soldier

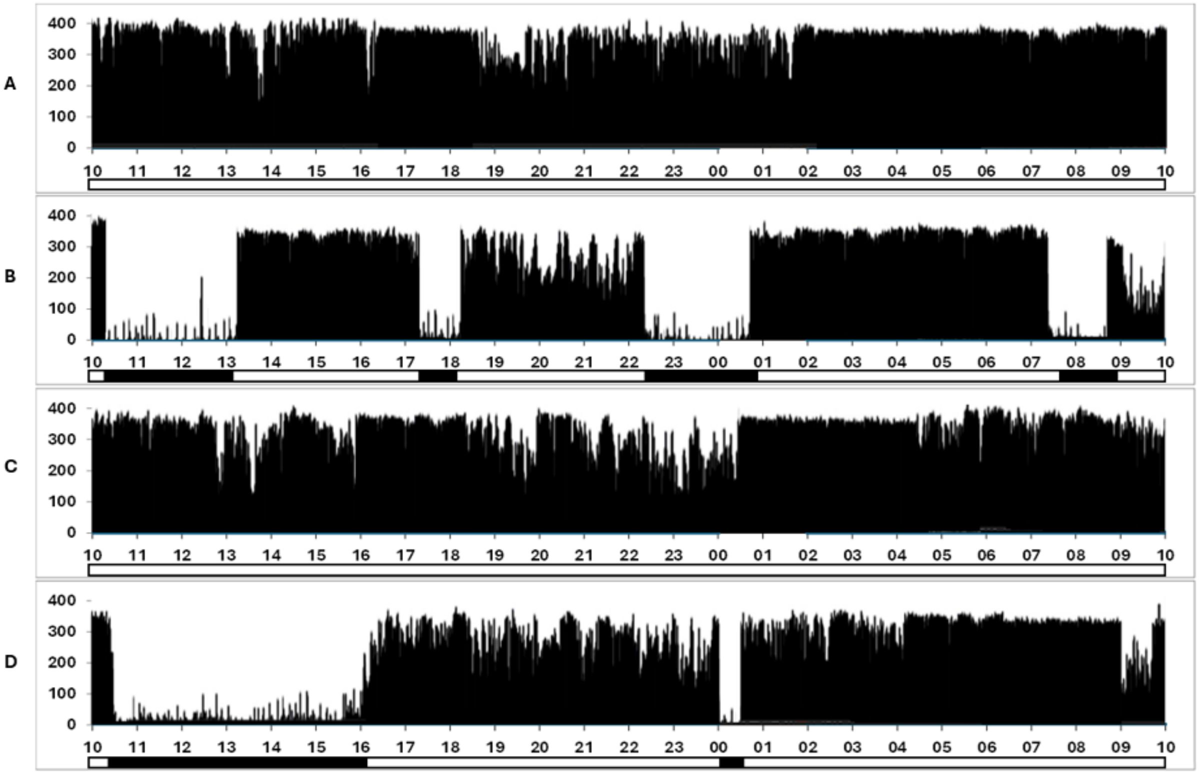

Figure 1. Representative actigraph data from one SF soldier was collected continuously over the 96-hour exercise. Panels A-D correspond to a single 24-hour period and plot the frequency of movements against time, measured in 60-second epochs. The y-axis quantifies movement counts, with the magnitude of the vertical line reflecting the detected movement count within each epoch. A secondary binary-coded axis beneath the x-axis indicates estimated sleep (solid bars) and wakefulness (clear segments) phases.

Table 1. Caffeine Consumption (mg) over the Previous 24 hours

Hours	Low (mg) (N=21)	High (mg) (N=7)
0	76.66±17.41	556.00±66.68
50	55.42±16.72	455.00±50.12
96	60.39±18.62	548.48±66.85
24-h average	64.32±10.18	525.56±43.79

Table 1. Caffeine consumption (mg) over the previous 24 hours at three time points (0, 50, and 96 hours) during a combat exercise. Data presented as mean ± SEM. Caffeine consumption 'Low' (<400 mg), and 'High' (≥ 400 mg).

Table 2. Cognitive Performance, Propensity to Take Risks, and Mood States to High and Low Caffeine Consumption During the 96-hour Exercise

Variable	0 h (Low/High)	50 h (Low/High)	96 h (Low/High)	F (df)	p-value	Partial η^2
**Psychomotor Vigilance Task**						
Mean Reaction Time (msec)	0.33±0.01/ 0.32±0.02	0.34±0.01/ 0.31±0.00	0.34±0.01/ 0.32±0.01	5.333 (1, 78)	0.024	0.064
Premature Responses (%)	6.31±1.16/ 5.14±2.49	9.60±1.58/ 4.02±1.40	10.16±1.47/ 3.89±2.21	5.137 (1, 78)	0.026	0.062
Timeout Responses (%)	4.84±1.15/ 8.55±4.84	8.51±1.36/ 3.49±1.26	9.20±1.47/ 3.90±2.21	1.396 (1, 78)	0.241	0.018
**Propensity to Take Risks**						
EVAR	12.86±0.05/ 14.76±0.31	11.93±0.10/ 12.92±0.64	12.02±0.07/ 13.31±0.56	4.494 (1, 78)	0.037	0.054
Self-Control	71.50±3.78/ 63.00±14.38	82.12±4.96/ 53.50±18.24	74.95±3.50/ 61.86±9.21	6.945 (1, 78)	0.01	0.082
Danger Seeking	52.92±2.53/ 34.00±4.14	62.76±3.30/ 59.00±8.55	60.14±3.71/ 59.00±7.69	5.649 (2, 78)	0.005	0.127
Energy	46.83±2.45/ 35.75±4.87	60.92±2.84/ 52.25±10.93	52.62±2.84/ 52.57±5.92	4.790 (2, 78)	0.011	0.109
Impulsiveness	86.58±3.57/ 83.50±6.44	88.08±4.18/ 86.00±6.88	92.29±4.02/ 87.71±8.56	0.302 (2, 78)	0.74	0.008
Invincibility	49.33±2.79/ 31.50±6.59	50.88±3.66 56.00±13.21	50.48±4.14/ 52.86±9.04	2.236 (2, 78)	0.114	0.054
**Profile of Mood States**						
Total Mood Disorder	95.67±2.33/ 95.75±4.94	117.29±2.15/ 119.00±6.70	106.19±1.65/ 108.57±3.32	16.867 (2, 78)	<0.001	0.302
Anger	1.54±0.60/ 0.25±0.25	5.21±0.74/ 7.75±3.07	1.29±0.44/ 3.00±1.45	12.050 (2, 78)	<0.001	0.236
Depression	2.08±0.65/ 1.75±1.03	5.33±0.96/ 4.00±2.45	1.52±0.47/ 2.43±1.57	2.751 (2, 78)	0.07	0.066
Fatigue	2.42±0.50/ 5.00±1.73	11.46±0.56/ 11.75±1.84	10.33±0.73/ 10.86±1.39	29.709 (2, 78)	<0.001	0.432
Tension	3.25±0.48/ 2.25±0.75	1.96±0.36/ 2.00±0.71	0.62±0.21/ 0.86±0.46	5.727 (2, 78)	0.005	0.128
Vigor	13.62±0.80/ 13.50±2.53	6.67±0.68/ 6.50±2.06	7.57±0.89/ 8.57±1.32	13.081 (2, 78)	<0.001	0.251
**Subjective Sleepiness**						
KSS	4.00±0.36/ 3.75±0.48	8.58±0.24/ 7.50±1.55	7.52±0.37/ 6.57±0.97	22.527 (2, 78)	<0.001	0.366

Table 2. The effect of high and low caffeine consumption on cognitive performance, propensity to take risks, Profile of Mood States, and subjective sleepiness during a combat exercise at 0, 50, and 96 hours. Data presented as mean ± SEM.
